# Complications in follicular unit excision hair transplantation: current evidence and practical approaches

**DOI:** 10.3389/fmed.2026.1750989

**Published:** 2026-02-03

**Authors:** Cristina Romera de Blas, David Vega Díez, José María Ricart Vayá, Alba Gómez Zubiaur

**Affiliations:** 1 Department of Dermatology, Hospital Universitario de Toledo, Toledo, Spain; 2 Trichology Unit, Dermatology, Instituto Médico Ricart, Madrid, Spain; 3 Department of Dermatology, Hospital Universitario Príncipe de Asturias, Alcalá de Henares, Spain

**Keywords:** complications, follicular unit excision, folliculitis, FUE, graft survival, hair transplantation, recipient-site necrosis, shock loss

## Abstract

**Introduction:**

Follicular Unit Excision (FUE) is currently the most widely used technique in hair transplantation due to its minimally invasive approach, rapid recovery and natural-looking results. Although generally considered a safe procedure, FUE is associated with a range of potential complications that may affect clinical outcomes and patient satisfaction. Recognizing and understanding these adverse events is essential to optimize patient safety and surgical success.

**Methods:**

We performed a comprehensive review of the literature, using PubMed and Embase databases, up to September 2025. Articles reporting FUE-related complications were selected and analyzed, focusing on incidence, pathophysiology, risk factors, clinical features, management, and prevention.

**Results:**

Complications were categorized into general postoperative events such as pain, edema, bleeding, and infection; donor area complications including hypopigmentation, hypertrophic scarring, epithelial cysts, and donor depletion; and recipient site complications such as necrosis, folliculitis, persistent perifollicular erythema, effluvium, and unnatural results. Less common but clinically relevant entities, including inflammatory and autoimmune reactions or atypical infections, were also reviewed. The development of complications is influenced by both patient-related factors such as comorbidities, smoking, or concurrent medications, and technical variables, including punch design, graft handling, follicular unit density, and ischemia time. Evidence-based strategies aimed at reducing complications and optimizing graft survival were reviewed.

**Conclusion:**

Although rare, complications following FUE can have significant implications. Careful patient selection, refined surgical technique, and standardized postoperative care are essential to reducing complication rates. This review offers a structured and evidence-informed framework for the identification, prevention, and management of complications in FUE.

## 1 Introduction

Hair transplantation is a well-established surgical procedure used to restore hair in areas affected by permanent alopecia. While androgenetic alopecia represents the primary indication, it is also performed for scarring-related hair loss (1, 2). Advances in technique and instrumentation in recent years have contributed to improved graft survival and more natural aesthetic results. Follicular Unit Excision (FUE) has become the predominant technique in hair transplantation surgery, surpassing follicular unit transplantation (FUT) because of its minimally invasive approach, faster postoperative recovery, and the absence of a linear donor scar.

Despite being widely regarded as a safe technique, FUE is not exempt from complications. Most are mild and self-limited, such as postoperative edema, pruritus, transient pain, or temporary effluvium, but others may compromise graft survival, delay healing, or affect the final cosmetic outcome (3).

Large clinical series estimate overall complication rates between 1.2 and 4.7%, with major adverse events being uncommon (4). Nonetheless, in the context of an elective cosmetic procedure, even minor complications can significantly affect patient satisfaction and may carry psychological or medico-legal implications. Reported risk factors include patient comorbidities such as diabetes or hypertension, smoking, and a predisposition to abnormal scarring, as well as technical aspects like inadequate surgical planning, poor graft handling, overharvesting of the donor area, excessive density, or suboptimal postoperative care (3, 5, 6). The growing number of procedures performed in poorly regulated settings has further contributed to preventable, iatrogenic complications (6). Therefore, an adequate preoperative assessment and careful patient selection are key to minimizing complications and ensuring optimal outcomes in hair transplantation.

Although several recent systematic reviews and meta-analyses have summarized complication data in hair transplantation, most combine heterogeneous surgical approaches and do not differentiate outcomes specific to FUE (2, 4). The available evidence therefore remains heterogeneous, with substantial variability in complication definitions and reporting. The objective of this review is to provide an updated, comprehensive narrative overview of current evidence focused exclusively on FUE, integrating data on incidence, risk factors, and practical management strategies to support clinical decision-making and optimize patient outcomes.

## 2 Methods

A narrative review of the literature was conducted to identify publications describing complications associated with FUE hair transplantation. PubMed and Embase databases were searched up to September 2025 using combinations of the following keywords: *hair transplantation, hair restoration, follicular unit excision, FUE, follicular unit extraction, complications, safety, pain, edema, swelling, effluvium, infection, erythema, necrosis, folliculitis, autoimmune complications*, and *graft survival*. Reference lists of included articles were also screened to identify additional relevant reports.

Articles were considered eligible if they reported complications related to FUE. Included studies consisted of retrospective cohort studies, case series, case reports, systematic reviews, meta-analyses, narrative and scoping reviews, and expert consensus statements. Publications focusing exclusively on FUT or on procedures unrelated to scalp hair transplantation were excluded.

All relevant studies were reviewed for data regarding incidence, risk factors, pathophysiology, clinical presentation, management, and prevention of FUE-related complications. Given the heterogeneity of study designs, definitions, and outcome measures, and the narrative scope of this review, no formal quality assessment or quantitative synthesis was performed.

## 3 Complications of follicular unit excision

Complications of FUE can be classified into general postoperative events (Table 1), donor-site complications (Table 2) and recipient-site complications (Table 3).

**Table 1:** General postoperative events.

**Complication**	Incidence^*^	**Timing**	**Key features**	**Risk factors**	**Prevention**	**Management**
Pain and sensory disturbances	~6%	First 24–48 h to day 3–5. Sensory changes may last days to few months.	Mild, self-limited discomfort, occasionally with transient hypoesthesia or hyperesthesia in donor or recipient areas.	Extensive donor harvesting, multiple punch excisions.	Adequate local anesthesia; atraumatic technique; patient counseling.	Non-opioid analgesics; reassurance.
Edema	~40%−50%	Onset day 1–2, peaks at days 2–3, resolves by 5–7 days.	Forehead and periorbital swelling, may be accompanied by mild ecchymosis.	Megasessions; dense frontal packing; excessive tumescence.	Careful tumescence; head elevation; perioperative corticosteroids (systemic or intratumescence); early cold compresses.	Conservative measures: head elevation, intermittent cold compresses; reassurance.
Bleeding	–	Intraoperative; mild postoperative oozing first 24–48 h.	Usually intraoperative and mild; postoperative bleeding uncommon.	Bleeding disorders; anticoagulants/ antiplatelets; NSAIDs; supplements; hypertension; cicatricial alopecia.	Preoperative medication review; blood pressure control; epinephrine-containing tumescence.	Local compression; tranexamic acid; reassessment if persistent.
Pruritus	Common	Typically appears days 3–7, lasting days to weeks. Persistent cases may extend up to 6 months.	Itching during early healing phase.	Crusts; scalp dryness; seborrheic dermatitis; irritant cleansing.	Gentle postoperative washing; avoidance of irritants; adequate scalp hydration.	Saline sprays; emollients; cold compresses; antihistamines or mild topical corticosteroids if persistent.
Infection	< 1%	First 1–4 weeks.	Usually superficial folliculitis or cellulitis; rare viral reactivation.	Poor scalp hygiene, excessive crust formation, diabetes, immunosuppression.	Aseptic technique; early gentle cleansing; postoperative wound hygiene.	Topical antibiotics for localized infection; systemic antibiotics if extensive or symptomatic.
Patient dissatisfaction	–	Early postoperative period; improves by 6–12 months.	Often related to unrealistic expectations, density or temporary shedding.	Unrealistic expectations, unstable alopecia, donor-recipient imbalance, very young age, psychological factors.	Careful patient selection; thorough preoperative counseling; realistic goal setting.	Reassurance; structured follow-up; time for maturation. Secondary procedures after ≥12 months if required.
Hiccups	Exceptional	Intraoperative or early postoperative (first 24–48 h)	May result from phrenic nerve irritation.	–	–	Usually self-limited; chlorpromazine or baclofen if persistent.

^*^Incidence rates are approximate and derived from heterogeneous sources, including retrospective series, case-based studies, and systematic reviews; true population-level incidence remains uncertain.

**Table 2:** Donor-site complications.

**Complication**	Incidence^*^	**Timing**	**Key features**	**Risk factors**	**Prevention**	**Management**
Overharvesting	Variable	Evident weeks to months after surgery	Excessive extraction density causing visible thinning, “moth-eaten” appearance, windowing effect, or permanent donor depletion.	Inadequate donor assessment; high graft demands; repeated sessions in the same area; low baseline donor density; inexperienced teams.	Thorough donor evaluation; long-term planning; limit extraction density (10%−20% per session); uniform distribution of punches; tapering at the margins.	Limited reconstructive options; cosmetic camouflage (longer hair, scalp micropigmentation); selective redistribution using peripheral or body donor areas.
Hypopigmentation	Common	Appears weeks to months after surgery.	Small, round hypopigmented macules at punch sites, more visible in darker skin or short hair.	Larger punches (>1 mm); high-density extractions; darker skin phototypes; short hairstyles	Use small punches (0.8–0.9 mm), adequate spacing; avoid overharvesting.	Camouflage with longer hair; scalp micropigmentation or graft redistribution if indicated.
Abnormal scarring	Rare	Months after surgery.	Hypertrophic or fibrotic scarring, surface irregularities, keloid formation.	Personal or family history of abnormal scarring; darker phototypes; large punch size; excessive density or depth.	Identify high-risk patients; use the smallest effective punch; limit extraction density; consider test session in high-risk individuals.	Intralesional corticosteroids; silicone-based products; fractional laser; combined therapies (e.g., 5-FU, PDL/CO_2_ laser) in selected cases.
Inclusion cysts	Uncommon	Weeks to months post-op.	Small, firm nodules at punch sites or in the recipient area; may become inflamed or infected.	Incomplete follicular extraction; excessive punch depth; follicular transection; blunt instruments. In the recipient area, buried grafts or placement over a previously implanted FU.	Complete follicular unit extraction; avoid transection; sharp, well-aligned punches; correct angulation and depth.	Observation if asymptomatic; intralesional corticosteroids; incision and drainage with antibiotics if inflamed; surgical excision if persistent.
Donor-site effluvium	Uncommon	Onset 2–4 weeks; resolution by 3–6 months.	Transient patchy or diffuse shedding of residual hair in the donor area.	Excessive trauma; vascular compromise; high extraction density; excessive tumescence or vasoconstriction.	Maintain safe extraction density; minimize mechanical trauma; avoid excessive tumescence or high vasoconstrictor concentrations.	Reassurance; expectant management; adjuvant therapies such as minoxidil, LLLT, or PRP may accelerate regrowth.
“Vasculitis-like” lesions	Rare	First week, spontaneous resolution within 14 days.	Purpuric plaques with central necrosis surrounding punch sites, mimicking cutaneous small-vessel vasculitis.	Use of motorized micropunches; mechanical vascular injury; possible thermal damage.	Meticulous surgical technique when using motorized punches.	Reassurance and observation; no specific treatment required; avoid unnecessary diagnostic procedures.
Arteriovenous fistula/ pseudoaneurysm	Exceptional	Days to weeks post-op.	Pulsatile, compressible mass with thrill or bruit (AVF); painful pulsatile mass (pseudoaneurysm).	Deep punch insertion; injury to underlying vessels.	Controlled punch depth; adequate tumescence.	Doppler ultrasound or CT angiography for diagnosis; surgical ligation or endovascular embolization.

5-FU, 5-fluorouracil; FU, follicular unit; LLLT, low-level laser therapy; PRP, platelet rich plasma; CT, computed tomography.

^*^Incidence categories are descriptive and based on published retrospective series and clinical reports, as population-based estimates are not available for most complications.

**Table 3:** Recipient-site complications.

**Complication**	Incidence^*^	**Timing**	**Key features**	**Risk factors**	**Prevention**	**Management**
Folliculitis and pseudofolliculitis	~12%	Folliculitis: 1–4 weeks; pseudofolliculitis: around week 10.	Erythematous papules or pustules around grafts; sterile or infectious; pseudofolliculitis related to emerging hairs.	Megasessions; high implantation density; delayed washing; procedures performed during summer months; buried grafts; thick, coarse or curved hair shafts.	Adequate asepsis; careful graft placement; controlled density; early postoperative cleansing.	Gentle cleansing; warm compresses; topical antibiotics; oral antibiotics if extensive; topical corticosteroids or tetracyclines for sterile inflammatory forms.
Recipient-site necrosis	Rare	Immediate to early postoperative period (hours–days)	Ischemic discoloration, crusting, eschar formation; potential graft loss and scarring.	Smoking; diabetes; vascular disease; prior surgery or radiotherapy; scarring alopecia; dense packing; megasessions; excessive tumescence; high epinephrine concentration.	Careful patient selection; smoking cessation; avoid dense packing; low epinephrine concentration; avoid deep, large or overlapping slits; intraoperative monitoring. Adjunctive regenerative therapies may be considered in high-risk cases.	Early recognition; pause surgery if ischemia suspected; topical nitroglycerin; conservative wound care; delayed secondary transplantation if required.
Persistent erythema	Occasionally reported	>4 weeks post-op	Prolonged redness surrounding transplanted follicles; may be associated with shedding or reduced graft survival.	Folliculitis; delayed postoperative washing (>3 days)	Gentle technique; early washing (< 72 h); avoid excessive trauma; post-operative PRP may be considered.	Usually self-limited; topical anti-inflammatory agents if symptomatic; evaluate for folliculitis or dermatitis if persistent
Crusting	Very frequent (physiological)	2–10 days post-op	Scab formation around grafts; persistent (>10–14 days) or thick crusts may indicate delayed healing or ischemia/necrosis.	Inadequate washing; excessive exudate.	Early gentle cleansing (24–72 h); patient education and close postoperative follow-up.	Moist compresses; gentle scalp soaks; emollient gels to facilitate detachment when required. clinical evaluation if crusts persist beyond 2 weeks.
Recipient-site effluvium (shock loss)	0.15%−15%	2–8 weeks after surgery. Regrowth around 3 months.	Diffuse or patchy shedding of native hairs adjacent to grafts.	Female sex; older age; FPHL; high implantation density; local trauma; over-tumescence; psychological stress.	Avoid excessive implantation density and tumescence; gentle surgical technique; preoperative counseling.	Patient reassurance; topical/oral minoxidil; cosmetic camouflage if needed. Spontaneous regrowth expected.
Unnatural results	Variable	Months post-op.	Artificial appearance from poor hairline design, hair angulation, direction; density distribution; “pluggy”/tufted look.	Inadequate preoperative planning; surgical inexperience; failure to anticipate alopecia progression.	Thorough preoperative assessment; realistic expectation setting; gradual and irregular hairline design; appropriate graft selection and placement.	Graft removal or redistribution; camouflage with additional single-hair grafts; laser hair removal or electrolysis in selected cases.
Poor growth	Variable	6–12 months post-op.	Sparse or uneven regrowth	Follicular injury during harvesting or implantation; prolonged out-of-body time; dehydration; smoking; vascular disease; factor X.	Gentle graft handling; minimize ischemia time; maintain graft hydration; avoid compression and desiccation; precise punch and slit technique.	Expectant management; optimization of medical therapy; secondary transplantation if indicated.
Autoimmune/ inflammatory reactions (LPP, EPS)	Rare	Months to years post-op.	LPP: late-onset perifollicular erythema, pruritus, scarring alopecia. EPS: pustules and crusted lesions that heal with scarring alopecia.	Undiagnosed cicatricial alopecia; trauma-induced immune activation (Koebner phenomenon).	Careful preoperative clinical and trichoscopic evaluation.	Biopsy for confirmation when clinical suspicion is high (new-onset perifollicular erythema, symptoms, progressive loss of follicular openings); disease-specific medical management.

FPHL, female pattern hair loss; LPP, Lichen planopilaris; EPS, erosive pustulosis of the scalp.

^*^Incidence estimates are based on heterogeneous retrospective series and clinical reports; reported frequencies vary widely, and true population-level incidence is unknown.

### 3.1 General postoperative events

#### 3.1.1 Pain and sensory disturbances

Pain is one of the most common early complaints after FUE, although it is generally mild and self-limited, usually well-controlled with analgesics (3, 5). Discomfort tends to be more pronounced in the donor area, occasionally accompanied by transient hypoesthesia or hyperesthesia (3, 4). In a recent systematic review and meta-analysis, donor-site pain was identified as the most frequently reported early complication, with a pooled incidence of 6% in FUE, and showed a non-significant trend toward lower rates in FUE compared with FUT (2). Consistent with these findings, a small comparative study reported minimal postoperative pain from the first postoperative day and near-complete resolution by day 3, with no clear associations between pain intensity and demographic or surgical variables (7).

Sensory disturbances, most commonly hypoesthesia or paresthesia, and less frequently hyperesthesia or dysesthesia, are thought to result from transection of small cutaneous nerve fibers during multiple punch excisions. Symptoms are typically transient, but persistent cases lasting several months have been reported, with complete resolution usually occurring within 4–8 months (4, 7). In a large FUE series, Aksoz et al. (8) reported long-term numbness or paresthesia in approximately 2% of patients, mainly in the temporal or occipital areas. While most patients experience only mild numbness or tingling, some may report dysesthesias or burning discomfort; however, these manifestations are rare and usually resolve spontaneously (3, 5).

Most patients achieve adequate relief with non-opioid oral analgesics such as paracetamol, metamizole, or nonsteroidal anti-inflammatory drugs (NSAIDs), and the need for stronger medication is exceptional. In cases of persistent sensory symptoms, treatment may include regional infiltrations of local anesthetics and corticosteroids, oral gabapentin or pregabalin, or botulinum-toxin injections (5, 9). Preoperative counseling and reassurance are important, since even minor or temporary sensory changes can cause significant anxiety in the context of a cosmetic procedure.

#### 3.1.2 Edema

**Figure 1:**
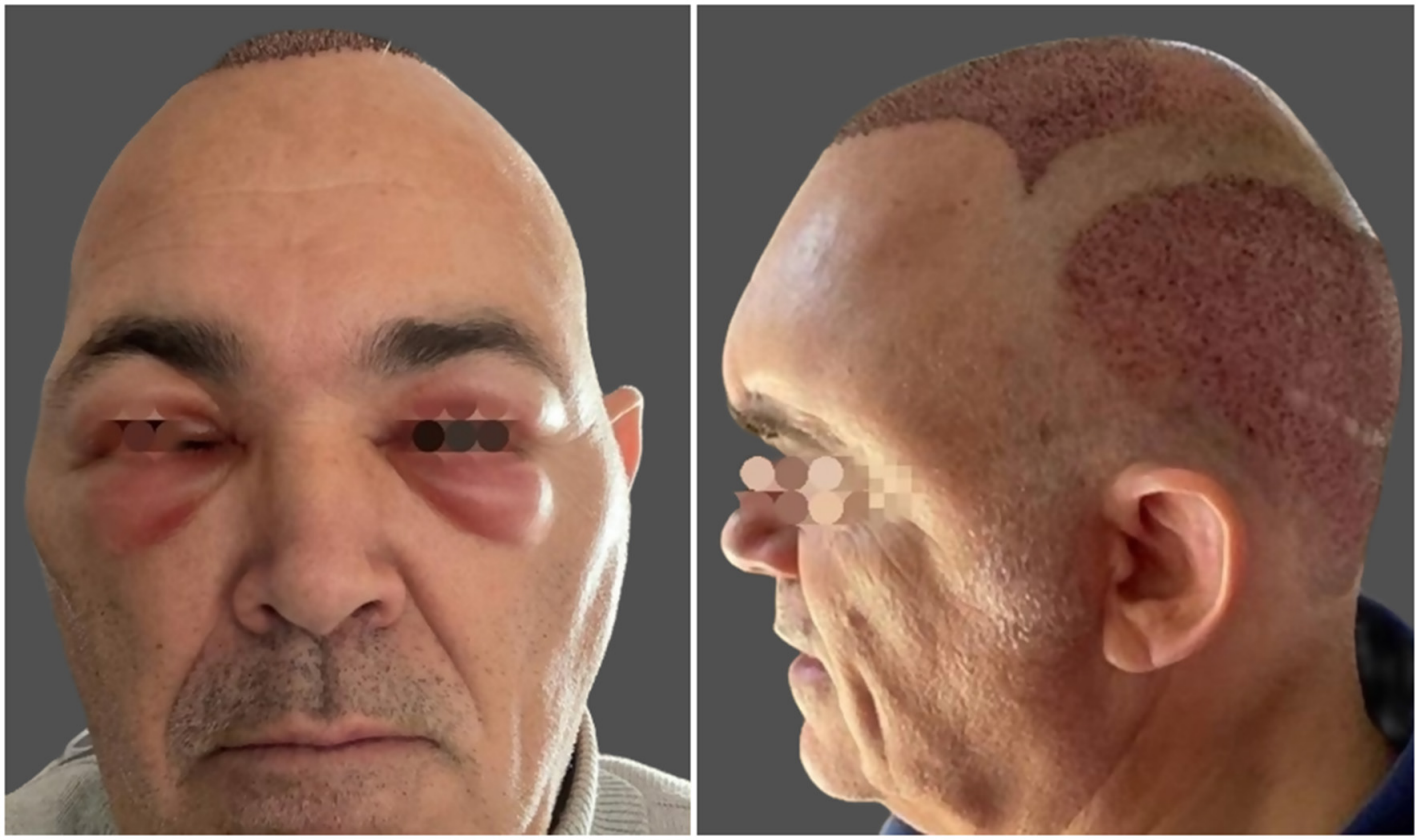
Frontal and periorbital edema at 48 h after FUE hair transplantation.

Edema is a common but generally minor postoperative event following FUE. It typically manifests as swelling of the forehead and periorbital tissues, peaking around 2–3 days after surgery and resolving spontaneously within 5–7 days (Figure 1) (1, 5). Although benign and self-limited, edema may cause temporary cosmetic or functional discomfort, which can be distressing for patients in the early recovery period. Mild ecchymosis may occasionally accompany edema, and in these cases its characteristic purple-yellow discoloration can persist for up to 10–15 days, which may be visually concerning for patients (5).

In FUE cohorts, frontal edema has been observed in nearly half of patients, making it the most common early postoperative finding, while periorbital swelling occurs in approximately 3%−5% (8, 10). Reported frequencies vary across publications, reflecting differences in tumescence volume, injection technique, postoperative positioning, and assessment criteria.

The pathophysiology involves infiltration of tumescent anesthesia and gravitational fluid shift toward the periorbital region, together with local inflammatory changes and transient lymphatic disruption. These effects tend to be more pronounced in megasessions or when dense packing is performed in the frontal scalp (1, 2, 4, 5).

Preventive measures include careful administration of the tumescent solution, perioperative corticosteroids (either intratumescence or short systemic courses), intermittent cold compresses and head elevation (20–30 °) during the first postoperative days (1, 5). In a prospective cohort, adding triamcinolone to the tumescent anesthetic solution reduced postoperative edema from 40 to 9% of cases (10). A recent multicenter study further demonstrated that, among patients receiving the same triamcinolone-containing tumescent solution and the same regimen of perioperative systemic corticosteroids, a preventive protocol using controlled subcutaneous injection and gentle compression after site creation to evacuate excess fluid significantly reduced both edema and ecchymosis compared with standard tumescence (11). Preoperative counseling remains essential to set expectations and reduce anxiety related to transient postoperative swelling.

#### 3.1.3 Bleeding

Bleeding may occur at any phase of hair transplantation, although it is usually mild and easily controlled with proper technique. Most cases are intraoperative and related to local vascular injury or elevated blood pressure, whereas postoperative bleeding is uncommon and typically self-limited (1, 12). Bleeding may also be more pronounced in primary cicatricial alopecias. Areas showing increased bleeding require close observation, as they may represent reactive ischemic points that could subsequently develop necrosis, or they may lead to the formation of thick hemorrhagic crusts that complicate postoperative care. Major hemorrhagic events are exceedingly rare in FUE procedures.

Preoperative evaluation, including screening for bleeding disorders and a detailed medication review, is essential. Oral anticoagulants, antiplatelet agents, NSAIDs, vitamin E, alcohol, and herbal supplements such as ginkgo biloba or ginseng can increase bleeding risk (6, 12). In patients receiving anticoagulant or antiplatelet therapy, the risks of bleeding and thrombosis should be assessed individually to determine whether temporary discontinuation is appropriate (13–15).

Intraoperative hemostasis is mainly achieved through adequate tumescence containing epinephrine at low concentration (1:100,000–1:450,000), which provides effective vasoconstriction without increasing the risk of ischemia. Maintaining stable blood pressure and good pain control further minimizes bleeding. In cases of significant bleeding, topical tranexamic acid can be used by adding one ampoule (5 ml, 500 mg) to the tumescent solution or by applying soaked gauze directly to the bleeding area. Oral or intravenous tranexamic acid may also be considered in selected patients (16–18). However, no comparative studies have defined the optimal route or dosing of tranexamic acid administration, and evidence regarding its potential thrombotic or necrotic risks remains limited.

Postoperative bleeding is infrequent and generally self-limited. Minor oozing can be managed by applying continuous pressure with a sterile gauze for 5–10 min, until hemostasis is achieved (12). Persistent bleeding despite adequate pressure should be reassessed clinically.

#### 3.1.4 Pruritus

Pruritus is a frequent complaint during the early healing phase in both the donor and recipient areas. It is usually mild, appearing within the first postoperative week and resolving spontaneously within several days or weeks (2–5). In a small proportion of patients, itching may persist for several months after surgery (8).

Pruritus is generally attributed to crust formation, scalp dryness, and epithelial regeneration. It tends to be more pronounced in patients with preexisting seborrheic dermatitis or in those using harsh cleansing agents after surgery. In cases of persistent or intense itching, underlying folliculitis, contact dermatitis, or an inflammatory reaction to topical antiseptics or hair care products should be ruled out.

Management is mainly supportive. Regular saline sprays and gentle cleansing help remove crusts and maintain hydration. Emollient or post-transplant moisturizing lotions can alleviate dryness, and cool compresses may provide additional symptomatic relief. For moderate or persistent pruritus, oral antihistamines and low-potency topical corticosteroids may be considered (3, 5). Patient education regarding gentle scalp care and avoidance of scratching is essential to prevent graft dislodgement and secondary infection.

#### 3.1.5 Infection

Postoperative infection after FUE is rare, with most studies reporting an incidence below 1%, although published rates vary across series. This low incidence is attributed to the rich vascular supply of the scalp and the minimally invasive nature of the technique (2, 4, 5). Predisposing factors include inadequate scalp hygiene, excessive crust formation, and preexisting medical risk factors such as diabetes or immunosuppression (19).

Typical clinical signs include pustules or honey-colored crusts, increasing pain, swelling, erythema, purulent drainage, and tenderness or enlargement of regional lymph nodes. These signs should be promptly recognized, and bacterial cultures with sensitivity testing obtained before starting antibiotic therapy.

Severe infections, occasionally leading to localized tissue necrosis, are uncommon but may jeopardize graft survival and aesthetic outcomes (20). Opportunistic infections caused by nontuberculous *Mycobacterium* spp. or *Mucor* spp. have been rarely reported, mostly after procedures performed in non-medical or poorly regulated settings (21, 22). Viral reactivations, particularly *Herpes simplex* infection and, in exceptional cases, Kaposi's varicelliform eruption, have also been described (23, 24).

Preventive strategies rely on strict intraoperative asepsis, early gentle cleansing, and proper postoperative wound care. Perioperative hygiene may also influence infection risk. The nasal vestibule is a common reservoir of *Staphylococcus aureus* and methicillin-resistant *S. aureus* (MRSA) in both patients and staff, and transmission during close-contact procedures has been documented (25). Although no specific guidelines exist for hair transplantation, decolonization measures such as intranasal mupirocin or preoperative antiseptic shampoo may be considered in selected high-risk cases or in practices with recurrent staphylococcal infections (5), including evaluation and treatment of colonized staff when necessary. Although many surgeons routinely prescribe oral antibiotics, current evidence does not support their systematic use in hair transplantation, and prophylaxis should, in most cases, be reserved for patients at increased risk of surgical-site infection.

Superficial infections usually respond to topical antibiotics such as mupirocin or clindamycin, whereas more extensive or diffuse infections with surrounding erythema, edema, or tenderness require systemic broad-spectrum antibiotics (12). Treatment should be initiated promptly and later tailored according to culture results. Early and appropriate therapy prevents graft compromise and promotes rapid recovery.

#### 3.1.6 Patient dissatisfaction

Patient dissatisfaction is among the most frequent non-medical complications after hair transplantation and may have psychological and medico-legal implications. Although most patients report high satisfaction, some may experience disappointment even when the surgery is technically successful (3, 5).

The main causes of dissatisfaction are unrealistic expectations, perceived insufficient density or delayed regrowth during the first postoperative months. Other contributors are suboptimal hairline design, ongoing native hair loss, limited donor supply, and preexisting psychological factors such as anxiety, body image concerns, or undiagnosed body dysmorphic disorder (12, 26). In a practice census by the ISHRS, 64% of men reported some degree of disappointment with density, underscoring the importance of expectation management (26).

Proper patient selection is fundamental to preventing dissatisfaction. Preoperative “red flags” such as unrealistic goals, unstable or rapidly progressive alopecia, significant donor–recipient imbalance, very young age with unpredictable long-term progression, or psychological vulnerability help identify candidates in whom surgery may not lead to durable or natural outcomes (27, 28). In such situations, deferring transplantation and prioritizing medical management may be the most appropriate course.

Equally important is comprehensive counseling on achievable density, the normal timeline of regrowth, the possibility of temporary shedding, and the limitations imposed by donor characteristics (1, 29). Expectations should extend beyond the first postoperative year, clarifying that alopecia may continue to evolve and that future procedures or ongoing medical therapy may be required to maintain natural coverage. Thorough documentation of informed consent ensures that patients understand these long-term considerations and helps reduce conflict (26, 28).

When dissatisfaction occurs, empathetic communication and structured follow-up are essential. Providing reassurance, addressing specific concerns, and allowing sufficient time for hair maturation usually resolve most cases. Secondary procedures or touch-up sessions should only be considered once final growth is achieved, typically around 12 months after surgery (12).

#### 3.1.7 Hiccups

Hiccups are an uncommon complication that may occur during or shortly after FUE hair transplantation. The underlying mechanism is not well-defined, but is thought to result from stimulation of the hiccup reflex arc, in which phrenic nerve involvement has been suggested (30, 31).

Episodes are typically self-limited; however, in cases where hiccups persist beyond the first 24 h, pharmacologic treatment may be considered. Chlorpromazine 25 mg orally two to three times daily or baclofen 10 mg orally every 6 h are effective therapeutic options (32).

### 3.2 Donor-site complications

#### 3.2.1 Overharvesting

**Figure 2:**
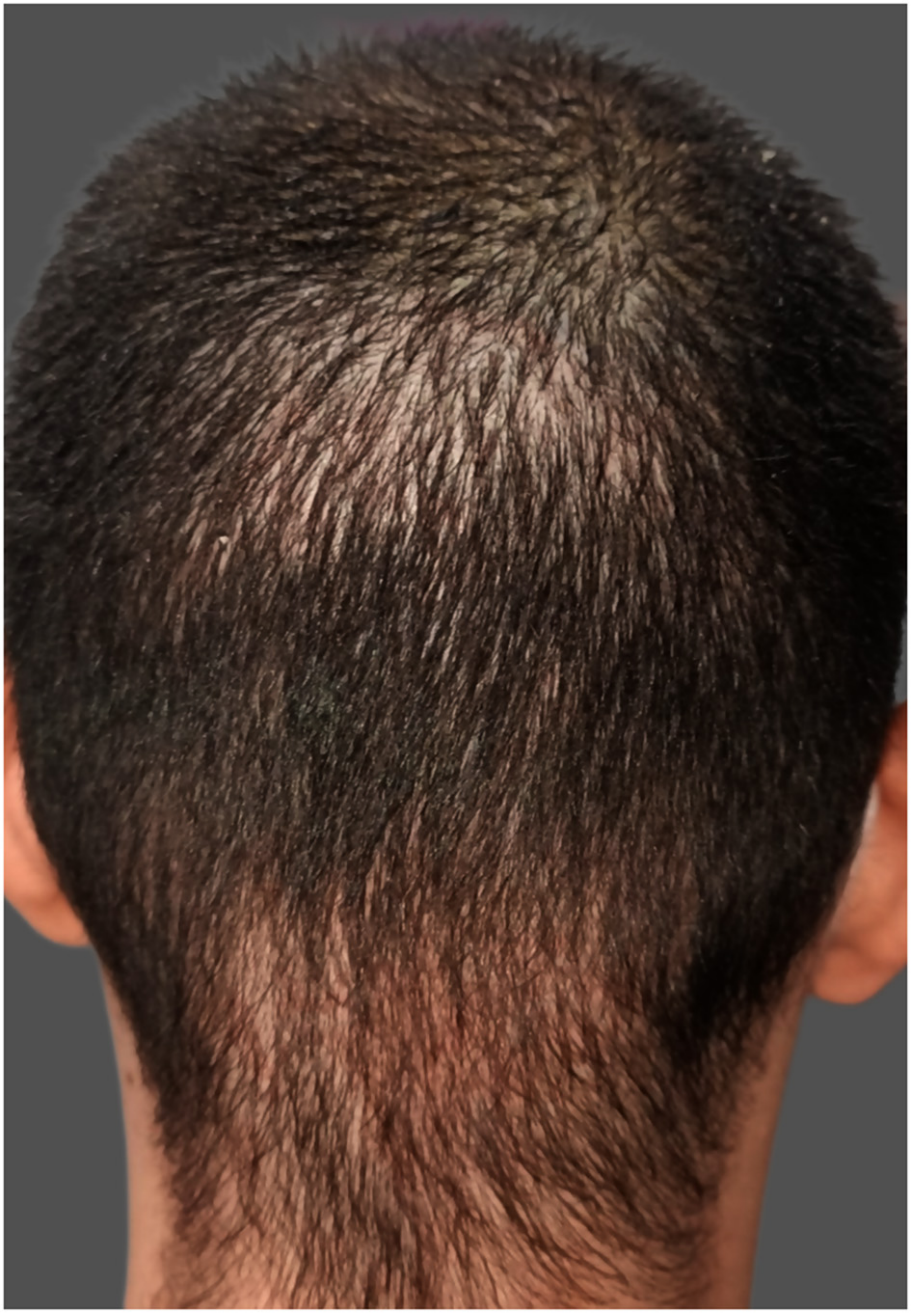
Window effect due to donor-site overharvesting.

Overharvesting is one of the most characteristic donor site complications in FUE procedures and is primarily associated with inadequate donor assessment or suboptimal surgical planning. It results from excessive extraction of follicular units from the donor area, leading to visible thinning, a “moth-eaten” appearance, “window” effect, or permanent donor depletion (Figure 2) (1, 5). This complication is increasingly observed in high-volume sessions exceeding 3,000–4,000 grafts, procedures performed by inadequately trained teams, and in patients requesting large graft counts despite insufficient donor density, often underestimating future balding progression (1).

The concept of safe donor area (SDA) refers to the occipital and parietal regions that tend to show greater long-term stability in androgenetic alopecia (33). However, its exact boundaries are not fixed and can vary considerably, especially in younger patients or in those with retrograde alopecia (27). Because of this variability, contemporary FUE practice often incorporates limited and carefully selected harvesting beyond the classical SDA when trichoscopy confirms low miniaturization and patients are counseled about long-term implications. This approach allows optimization of graft numbers while minimizing future instability.

**Figure 3:**
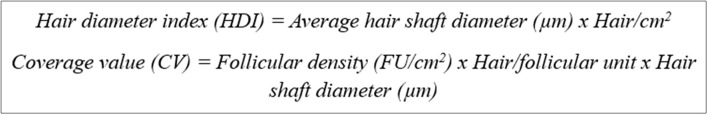
Formulas used to calculate the Hair Diameter Index (HDI) and Coverage Value (CV).

Donor evaluation should therefore combine anatomical mapping with quantitative assessment of follicular unit density, hairs per graft, shaft diameter, and miniaturization to ensure long-term stability and avoid overharvesting. Additional factors such as hair color, curl pattern, usual hair length, patient age, and anticipated androgenetic alopecia progression substantially influence perceived density and the cosmetic impact of extraction (1). Some authors have proposed indices such as the Hair Diameter Index (HDI) and Coverage Value (CV) (Figure 3), which integrate hair shaft caliber and follicular unit density to provide a more individualized estimation of the donor area's visual coverage potential (1, 34, 35).

Although no standard extraction limit has been established, many authors recommend limiting extractions to 10%−20% of the baseline follicular unit density per session, ensuring adequate spacing and distribution (36). Exceeding this range, particularly in low-density scalps, may compromise dermal vascularity, delay healing, and result in visible thinning even when scarring is minimal (1, 3).

Preventive strategies rely on thorough preoperative evaluation of the donor area using trichoscopy or digital dermoscopy, combined with long-term planning aligned with the expected pattern of hair loss. Uniform distribution of extractions, limiting the number of grafts harvested per cm^2^, and avoiding repeated sessions in the same zone are essential to preserve donor integrity (1, 37). To prevent sharp transitions or “window effect” at the borders of the extraction zone, a tapering or gradient pattern of extractions at the margins can help create a more natural appearance. Once donor depletion has occurred, reconstructive options are limited. Scalp micropigmentation or carefully planned redistribution of grafts from peripheral or body donor areas may help improve aesthetic outcomes (1, 38).

#### 3.2.2 Hypopigmentation

**Figure 4:**
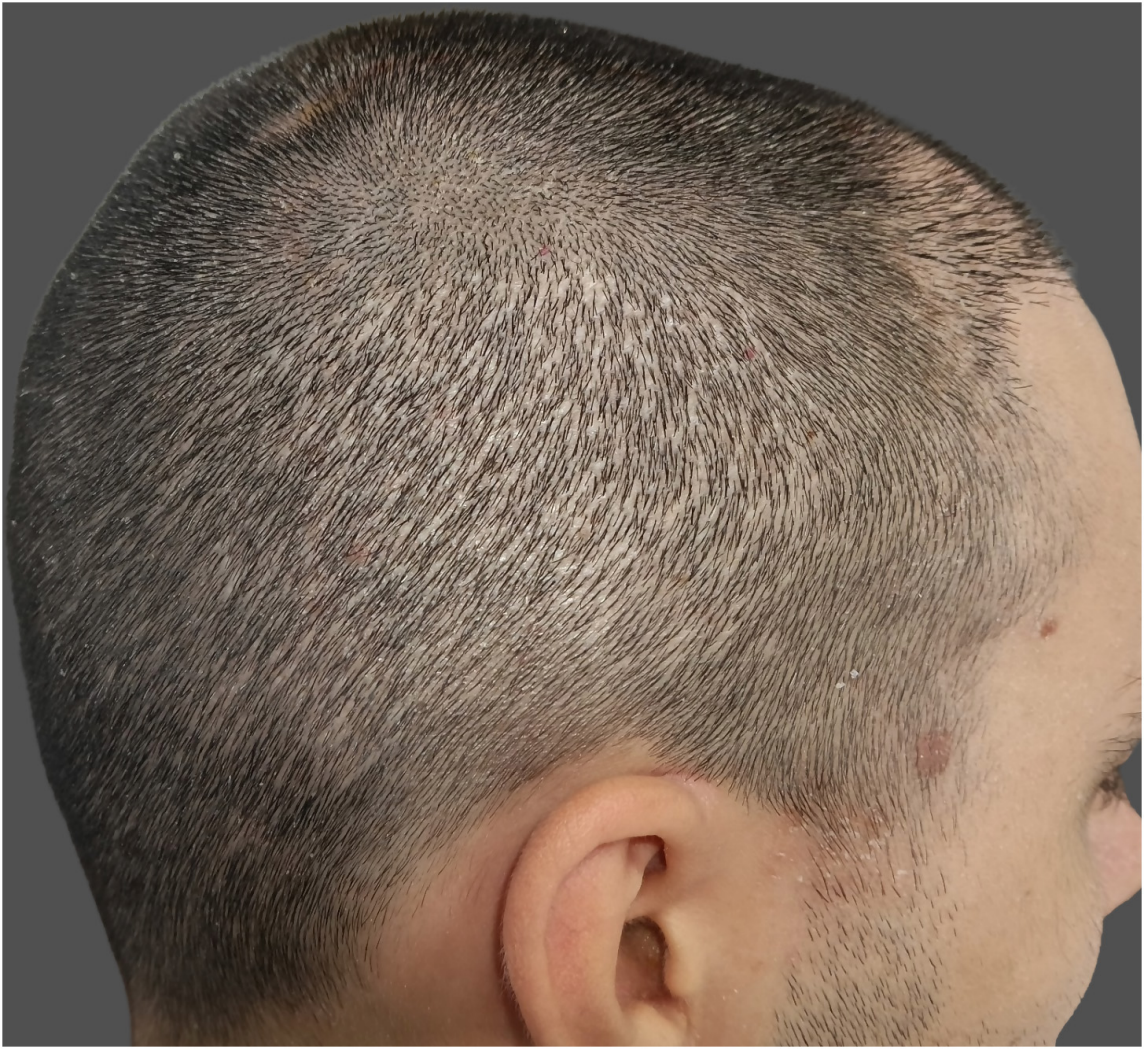
Pinpoint white dots in the donor-site area.

Hypopigmentation, commonly referred to as “pinpoint white dots,” is a recognized donor-site sequela of FUE characterized by small, round, depigmented macules at extraction sites. It is a misconception that FUE is a scarless technique; although it avoids linear scarring, each punch leaves behind a small wound that heals with some degree of fibrosis, often imperceptible but occasionally manifesting as visible hypopigmented spots (Figure 4). Hyperpigmentation has been less frequently reported. These atrophic scars tend to be more noticeable in patients with dark hair, darker skin phototypes, or those who prefer short hairstyles (5, 39).

The underlying mechanism involves transepidermal removal of follicular units, which eliminates not only the hair bulb but also follicular melanocytes, the principal reservoir of epidermal pigment cells. Larger punches (>1 mm) and high-density extractions increase dermal disruption and reduce melanocyte repopulation during healing, resulting in more noticeable hypopigmented scarring (3, 5). Inadequate postoperative wound care or delayed re-epithelialization may further accentuate the contrast between scarred and surrounding skin.

Prevention relies primarily on surgical technique optimization. Using smaller punches (0.8–0.9 mm), maintaining a uniform extraction pattern with densities below 15%−20% per session, and respecting adequate spacing between excisions are essential to minimize visible scarring. Patients at higher risk should be appropriately counseled preoperatively about the potential visibility of donor scarring, particularly if they plan to shave their scalp (5, 37).

Management focuses on improving visual appearance. Mild cases often improve as the scalp repigments over time or with hair growth coverage. In persistent or aesthetically significant cases, scalp micropigmentation may offer effective camouflage, and in selected cases, follicular redistribution from peripheral or body donor areas may improve coverage (1). Fractional ablative lasers, although not widely studied in this setting, may offer theoretical benefits by remodeling scar texture and promoting repigmentation (40, 41).

#### 3.2.3 Abnormal scarring

Abnormal scarring in the donor area after FUE is rare, especially when compared to the linear scarring associated with FUT. When present, it may manifest within the first few months after surgery as hypertrophic scars, fibrotic tracts, keloid scarring, or surface irregularities around punch sites (3, 12). Although keloidal scarring is exceedingly uncommon and typically limited to case reports, diffuse fibrosis or thickening may occur in some patients, particularly when large punches are used, extraction density is excessive, or spacing between punches is inadequate (42–44).

The pathogenesis involves an aberrant wound-healing response characterized by excessive fibroblast proliferation, collagen deposition, and prolonged inflammation, often triggered by local trauma, infection, or delayed re-epithelialization (3, 42). Risk factors include a personal or family history of abnormal scarring, darker skin phototypes, and previous keloid formation (45). In high-risk individuals, a small test session with limited harvesting is advisable to assess the patient's scarring response before full surgery (46). For the same reason, FUE using body or beard hair should be avoided unless a preliminary test extraction confirms a favorable healing pattern.

Prevention focuses on using the smallest effective punch, limiting extraction density, and identifying high-risk patients. Management depends on severity and clinical presentation. Early hypertrophic changes may improve with intralesional corticosteroids, silicone-based products, or fractional laser therapies. More persistent lesions may require combined approaches, including triamcinolone acetonide (10–40 mg/ml) injections, 5-fluorouracil, bleomycin, silicone sheeting, cryotherapy, imiquimod, and occasionally pulsed-dye or fractional CO_2_ laser treatment (47). Platelet-rich plasma has also been explored for its potential role in modulating scarring and promoting tissue repair (42). Surgical excision is rarely indicated, given the high risk of recurrence.

#### 3.2.4 Inclusion cysts

Epidermal inclusion cysts are uncommon complications following FUE, typically resulting from incomplete extraction of follicular units or inadvertent implantation of epidermal fragments into the dermis during punch excision (3, 5). Clinically, they present as small, firm, occasionally mobile nodules in the donor area, appearing weeks to months after surgery. While most remain asymptomatic and are discovered incidentally, some may become inflamed or secondarily infected, presenting with erythema, tenderness, or purulent drainage.

The underlying mechanism involves downward displacement of keratinizing epithelium that continues to produce keratin, leading to the formation of a cystic cavity. Risk factors include excessive punch depth, follicular transection, blunt instrumentation, improper angulation, and over-tumescence (6). Cysts may also develop in the recipient area, where they are more common, typically arising when a graft is inadvertently buried or inserted over a previously placed follicular unit, potentially compromising local healing and graft survival (12).

Prevention is primarily technical. Ensuring complete follicular unit extraction, avoiding transection of the upper follicle, and using sharp, properly aligned punches at the correct angle relative to hair direction are key preventive measures (6).

Management depends on severity. Asymptomatic lesions may be observed or treated with intralesional corticosteroids. Painful or inflamed cysts are managed with incision and drainage, along with topical or systemic antibiotics when infection is present. Persistent or recurrent lesions may necessitate complete surgical excision (6).

#### 3.2.5 Donor-site effluvium

**Figure 5:**
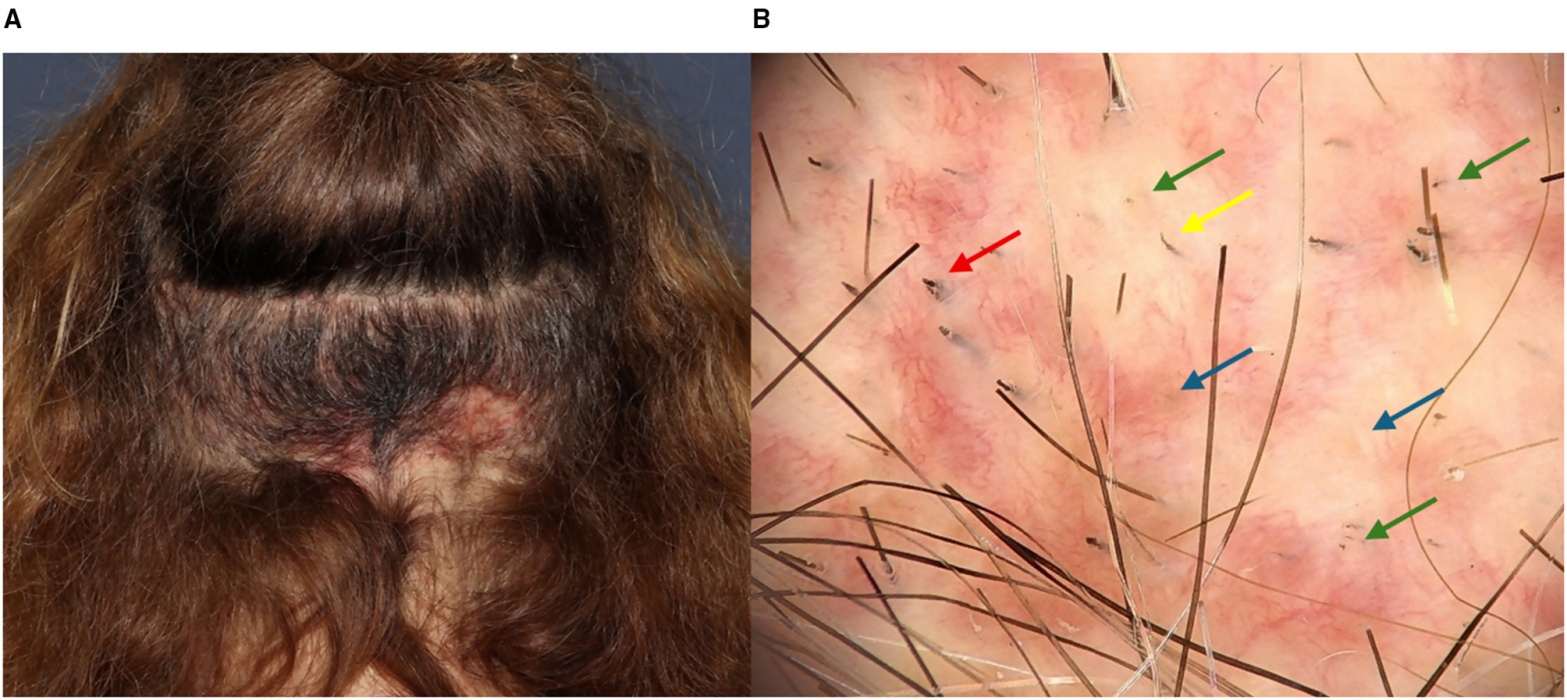
**(A)** Patchy presentation of donor-site effluvium. **(B)** Trichoscopic findings showing empty follicular ostia (blue arrow), short regrowing hairs (red arrow), dystrophic hairs (yellow arrow), and black dots (green arrow).

Donor-site effluvium is a transient shedding of residual hairs within or adjacent to the extraction area. It typically develops 2–4 weeks after surgery and resolves spontaneously within 3–6 months (48, 49). The true incidence remains unknown, as only isolated case reports and small series have been published. Overall, donor-site effluvium appears considerably less frequent than recipient-site effluvium (48–51). While recipient-site effluvium tends to be diffuse, donor-site effluvium often presents as a more localized thinning patch that can clinically resemble alopecia areata (Figure 5) (49).

The pathogenesis is multifactorial, involving local trauma, transient vascular compromise, and perifollicular inflammation that temporarily interrupt the hair cycle (50, 52). The exact phase affected remains debated, although traditionally interpreted as a localized telogen effluvium, the early onset after surgery and trichoscopic findings such as black dots and dystrophic hairs have led some authors to propose an acute anagen effluvium-like mechanism (49, 52). Current interpretation suggests that donor effluvium likely represents a spectrum of stress-induced shedding with features overlapping both phases, rather than a pure telogen process.

Clinically, donor-site effluvium presents with preservation of follicular openings and absence of scarring. Trichoscopy reveals empty ostia, short regrowing hairs, coudability hairs, black dots or dystrophic hairs (48, 49, 51). A differential diagnosis to consider is alopecia areata. Although trichoscopic findings such as black dots and broken or dystrophic hairs may overlap with alopecia areata or trichotillomania, donor effluvium follows a postoperative timeline, shows a negative pull test, is confined to the donor zone, and demonstrates spontaneous regrowth within weeks to months (48, 49). These features, together with the absence of response to intralesional corticosteroids, help differentiate it from true alopecia areata.

Management is primarily conservative. Patient reassurance is essential, as spontaneous regrowth is expected in most cases (4). Adjuvant therapies such as minoxidil, low-level laser therapy, and platelet-rich plasma may help accelerate hair regrowth (48, 49). Preventive measures include maintaining a safe extraction density, avoiding excessive tumescence or high vasoconstrictor concentrations, and minimizing mechanical trauma during harvesting.

#### 3.2.6 “Vasculitis-like” lesions

**Figure 6:**
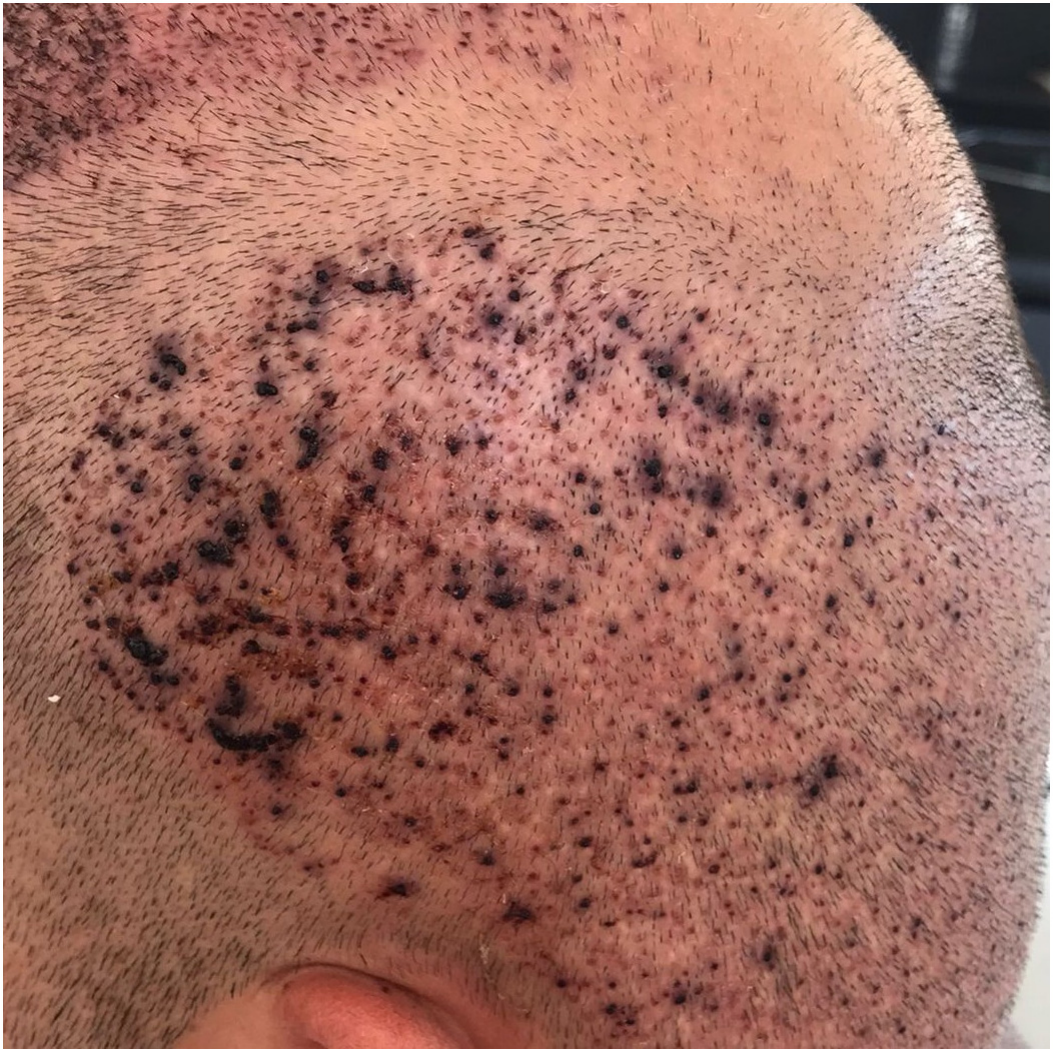
“Vasculitis-like” purpuric lesions in the donor area following follicular unit excision.

A recently described and rare donor-site reaction is the appearance of “vasculitis-like” purpuric lesions following follicular unit excision (53). Mir-Bonafé et al. reported a multicenter series of 10 patients who developed asymptomatic purpuric plaques with a necrotic center surrounding punch sites within the first postoperative week after extraction with motorized micropunches. All lesions resolved spontaneously, without sequelae, within 14 days. Lesions ranged from single to multiple, with diameters varying from a few millimeters to approximately two centimeters, and were strictly confined to areas of follicular extraction, clinically mimicking cutaneous small-vessel vasculitis (Figure 6) (53).

Trichoscopy revealed violaceous lesions with central necrosis, while histopathology demonstrated dermal hemorrhage without evidence of true vasculitis or thrombosis. The authors proposed mechanical vascular injury related to motorized punch movements, potentially combined with thermal damage, as the most plausible pathogenic mechanisms (53). Although clinically alarming, this entity appears to be benign and self-limited, and its recognition is important to reassure patients and avoid unnecessary diagnostic procedures or treatments.

#### 3.2.7 Arteriovenous fistula and pseudoaneurysm

Arteriovenous fistula (AVF) is a serious but extremely rare donor-site complication following FUE. It results from simultaneous injury to an artery and an adjacent vein during deep punch insertion, leading to the formation of an abnormal arteriovenous communication. Clinically, AVF presents as a pulsatile, compressible swelling with a palpable thrill or audible bruit, typically developing days to weeks after surgery (1, 54, 55). In a recent systematic review and meta-analysis, 11 donor-site AVFs and 2 recipient-site AVFs were identified among published reports (2). Although the true incidence cannot be precisely established, available data suggest a frequency well below 0.1%.

Diagnosis is confirmed by Doppler ultrasonography or CT angiography, which demostrate abnormal arteriovenous flow. Management generally requires surgical ligation or endovascular embolization (54, 56, 57). Preventive measures include controlled punch depth and adequate tumescence to increase dermal thickness and protect underlying vessels (1).

Other vascular complications, such as pseudoaneurysm formation, have also been reported but are exceedingly rare (58, 59). Pseudoaneurysms result from partial arterial wall laceration and present as painful, pulsatile, non-compressible masses appearing days to weeks postoperatively. Management consists of surgical ligation or endovascular embolization.

### 3.3 Recipient-site complications

#### 3.3.1 Folliculitis and pseudofolliculitis

**Figure 7:**
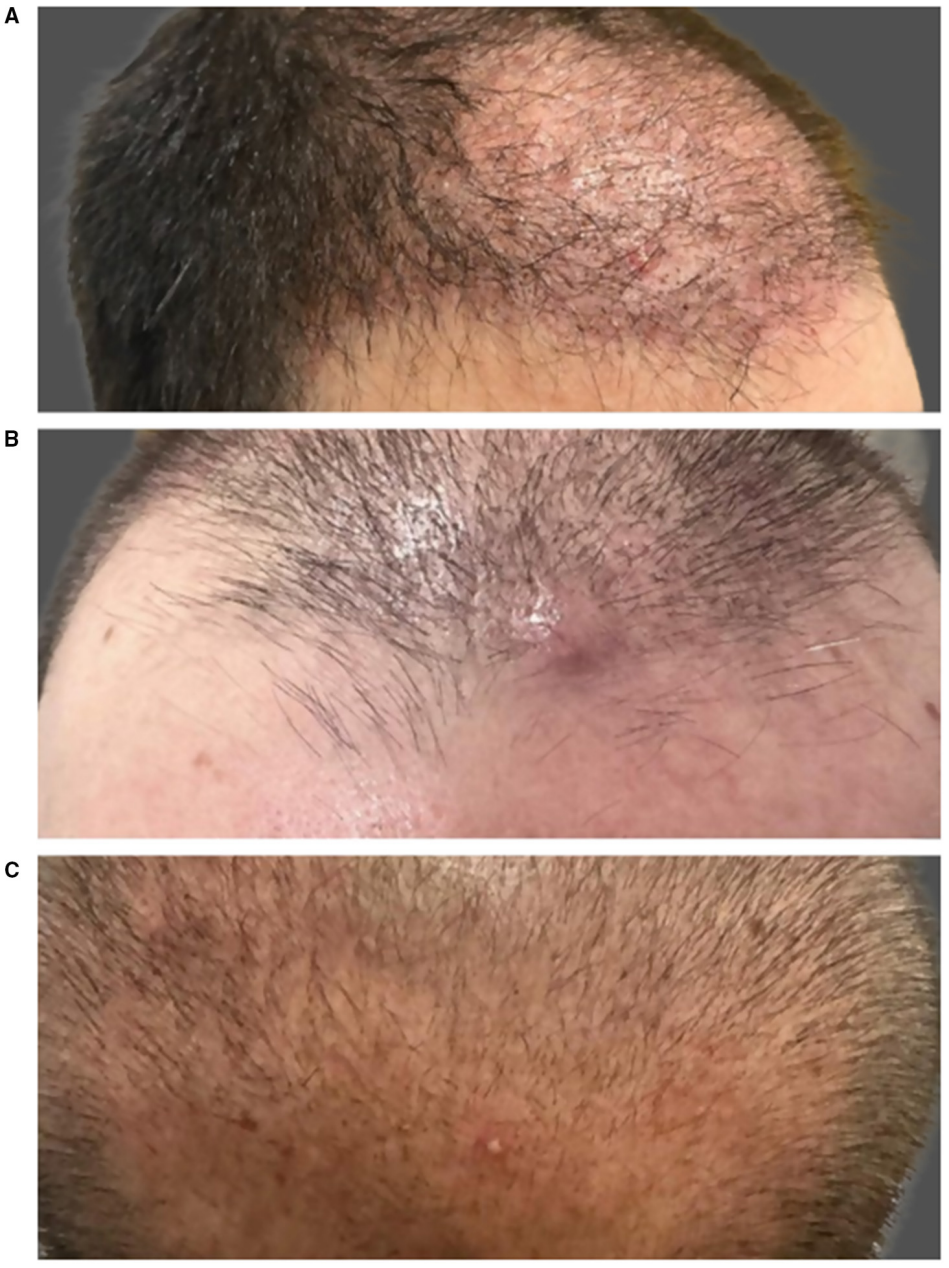
Clinical presentations of sterile folliculitis in the recipient area **(A–C)**.

Folliculitis is one of the most frequently reported postoperative complications following FUE. In a large multicenter retrospective study, Zhou et al. reported an incidence of 12.1%, consistent with prior literature. Donor-site involvement was observed in 46.7%, recipient-site involvement in 40.3%, and both areas were affected in approximately 13% of cases. The onset typically occurs within the first 1–4 weeks after surgery, manifesting as erythematous papules or pustules surrounding follicular units, occasionally accompanied by mild pain or pruritus (Figure 7) (60).

Pathogenesis is likely multifactorial. In the study of Zhou et al., (60) the risk of folliculitis was significantly higher in megasessions exceeding 4,000 grafts, high implantation density (>45 FU/cm^2^), delayed postoperative washing (>3 days), and procedures performed during summer months. Other authors have proposed that mechanical factors such as buried grafts, improper implantation angle, or excessive pressure during graft placement may also contribute (5, 61).

Folliculitis can be classified into infectious and sterile (inflammatory) folliculitis. Infectious folliculitis, usually caused by *Staphylococcus aureus*, typically appears early, whereas sterile folliculitis tends to develop later in the postoperative period and is characterized by perifollicular inflammation without detectable microbial growth (60). Sterile folliculitis includes both acneiform inflammatory reactions and pseudofolliculitis, a mechanical irritation caused by newly growing hairs. Pseudofolliculitis usually emerges around week 10, coinciding with early graft growth, and is particularly common in patients with thick, coarse, or tightly curved hair shafts, in whom the growing hair may curve back toward or irritate the epidermis.

Management is primarily conservative. Gentle cleansing and warm compresses help to promote crust removal and facilitate drainage of superficial pustules. Topical antibiotics, such as mupirocin or clindamycin, represent first-line therapy for localized lesions, whereas oral antibiotics are reserved for more extensive or painful cases. Persistent or sterile inflammatory folliculitis may benefit from short courses of topical corticosteroids or oral tetracyclines for its anti-inflammatory effect. Surgical drainage is rarely required (4, 8, 62, 63).

Preventive measures include proper intraoperative asepsis, careful graft handling to avoid transection, controlled implantation density, and early postoperative cleansing within 48–72 h after surgery. Routine antibiotic prophylaxis is not universally recommended but may be considered in megasessions or in patients with additional risk factors. The overall prognosis is favorable, as most cases resolve without sequelae; however, extensive or recurrent folliculitis may delay hair regrowth and negatively affect graft survival (60). Because pseudofolliculitis represents a later-onset mechanical reaction, additional monitoring during the period when early graft growth begins may be useful, particularly in individuals with inherently thick or coarse hair shafts.

#### 3.3.2 Recipient-site necrosis

Recipient-site necrosis is a rare but potentially severe complication of hair transplantation, characterized by partial or complete tissue loss at the implantation site, potentially leading to graft failure and scarring. Its true incidence remains unknown, as only isolated case reports and small series have been published to date (20, 64–66).

Recipient-site necrosis results from impaired perfusion and subsequent ischemia of the implanted area. Pathogenesis is multifactorial, involving both patient-related and surgical factors. Systemic conditions such as smoking, diabetes, hypertension, and peripheral vascular disease can compromise scalp microcirculation and delay wound healing. Locally, prior surgery or radiotherapy, scarring alopecia, or thin atrophic skin may predispose to ischemia. Technical risk factors include dense packing (>50 FU/cm^2^), megasessions (>3,500–4,000 grafts), deep or overlapping slits, excessive tumescence, and high concentrations of epinephrine (9, 20, 64, 65, 67). The mid-scalp region appears to be the most frequently affected, due to its relatively lower vascular supply (20, 64). Feily et al. (64) reported a right-sided predominance of necrosis, although subsequent scalp flowmetry studies have not confirmed vascular asymmetry between hemispheres.

**Figure 8:**
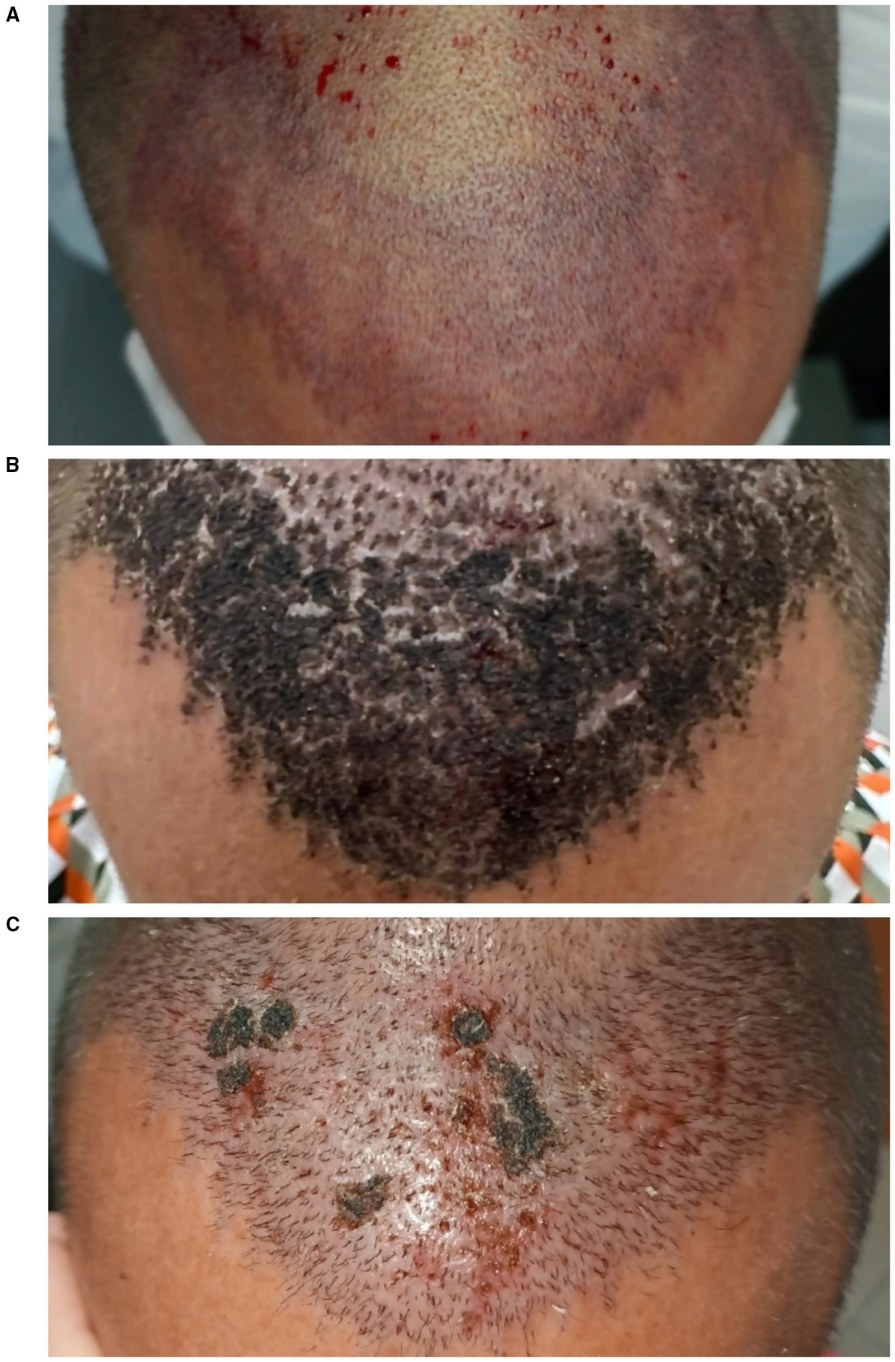
Clinical evolution of recipient-site necrosis. **(A)** Intraoperative blanching and violaceous discoloration after slit creation. **(B)** Day 7: formation of thick, dark crusts over the affected area. **(C)** Day 14: partial crust detachment with superficial erosions corresponding to necrotic tissue loss.

Early signs of impending necrosis may appear intraoperatively as blanching after local anesthesia or tumescence and as violaceous discoloration following slit creation. In the early postoperative period, dark discoloration, serous or purulent exudate, and the subsequent formation of black eschars or thick crusts have been described. After crust detachment, erosion or ulceration may be observed, which can compromise follicular unit survival within the affected area (Figure 8). The proportion of surviving follicles is influenced by the extent and depth of necrotic injury, with deeper involvement carrying a higher risk of permanent loss.

There is no standardized management protocol. Early recognition is essential to prevent progression. When vascular compromise is suspected intraoperatively, surgical manipulation in the affected area should be paused until perfusion improves (64). Topical nitroglycerin 2% ointment has been described as an initial measure to promote vasodilation when early signs such as pallor or cyanosis appear (67). Once necrosis is established, management is largely conservative, consisting of gentle debridement of devitalized tissue, maintenance of a moist wound environment, and topical antibiotics to minimize local bacterial load and facilitate separation of the overlying scab. Systemic antibiotics may be added if secondary infection is suspected. Healing may require several weeks to months and can result in atrophic or fibrotic scarring (20, 65). In cases with decreased graft survival, secondary transplantation can be considered at least 6 months after complete wound healing (20).

Preventive strategies aim to minimize ischemic risk through careful patient selection and technical optimization. Smoking cessation for at least 1 month before surgery, control of metabolic comorbidities, and preoperative assessment of high-risk areas such as scarred or previously operated regions are essential (20). Autologous fat grafts, exosomes, PRP injection and topical minoxidil treatment may improve the wound-healing capacity and blood supply (20). Intraoperatively, prevention relies on maintaining moderate implantation densities (< 50 FU/cm^2^), using low epinephrine concentrations (< 1:200,000), and avoiding excessive tumescence, deep, large or overlapping slits, and prolonged operative times (20, 67). Continuous intraoperative monitoring for color changes or blanching is recommended to detect early vascular compromise (67).

#### 3.3.3 Persistent erythema

Recipient-area perifollicular erythema (RPE) is a common and expected finding during the early postoperative period, reflecting the inflammatory and angiogenic phases of wound healing. In most patients, it resolves spontaneously within 2–4 weeks. However, a subset of individuals develops persistent RPE, characterized by redness surrounding transplanted follicular units that may last for several weeks or months.

In a recent multicenter cohort of 1,090 patients, Zhang et al. reported mild RPE in 16.3%, moderate in 5.1%, and severe in 0.9% of cases. More intense RPE tended to persist longer and was associated with increased postoperative shedding and reduced graft survival. Folliculitis and delayed postoperative washing (>3 days) were identified as independent risk factors for moderate to severe cases. Other proposed mechanisms include persistent perifollicular inflammation, microbial imbalance, and local immune dysregulation compromising follicular immune privilege (68).

Management is generally conservative. Gentle cleansing and early washing within the first three postoperative days are recommended to minimize bacterial overgrowth and inflammation. Prophylaxis with topical antibiotics is not routinely recommended but may be considered in selected high-risk patients. Postoperative platelet-rich plasma (PRP) injections have been proposed to reduce inflammation and promote healing, as well as graft growth. Prospective studies are needed to define standardized preventive and treatment strategies (68). Although RPE is usually self-limited, persistent erythema should be assessed for folliculitis, infection, or irritant/contact dermatitis.

#### 3.3.4 Crusting

Crust formation is a normal part of postoperative healing after FUE, typically appearing within 2–3 days and resolving spontaneously by 7–10 days with appropriate care. Delayed scab detachment may increase the risk of perifollicular inflammation or folliculitis, often related to inadequate washing or premature manipulation. Early gentle cleansing with saline or mild shampoo within 24–72 h promotes faster re-epithelialization and reduces bacterial colonization (4, 12, 69).

Persistent or thick scabs can delay re-epithelialization and sustain local inflammation, potentially compromising graft growth (12). In contrast, the early appearance of thick crusts accompanied by increased exudate should raise suspicion of ischemic injury or evolving recipient-site necrosis (20). Persistent crusts beyond 2 weeks should therefore be evaluated, as they may indicate delayed healing. Moist compresses, gentle scalp soaks, or topical emollient gels may help facilitate separation of adherent scabs when required (12). Close postoperative follow-up is important to ensure appropriate crust detachment, with progressive adjustments in washing technique and pressure as re-epithelialization progresses.

#### 3.3.5 Recipient-site effluvium (“shock loss”)

Recipient-site effluvium, commonly referred to as “shock loss,” denotes the transient shedding of native hairs surrounding the implanted area. It typically occurs within 2–8 weeks after surgery, with regrowth usually beginning around 3 months postoperatively. Reported incidence varies widely, ranging from 0.15 to 15% (70). Female sex has been identified as a major risk factor, with older age further increasing susceptibility among women with female pattern hair loss (FPHL) (70).

The mechanism is multifactorial, involving transient disruption of the hair cycle secondary to surgical trauma, perifollicular inflammation, local ischemia, or the use of vasoconstrictive agents such as epinephrine. High implantation density (50–70 FU/cm^2^), over-tumescence, and psychological stress have also been proposed as contributing factors (12, 71).

**Figure 9:**
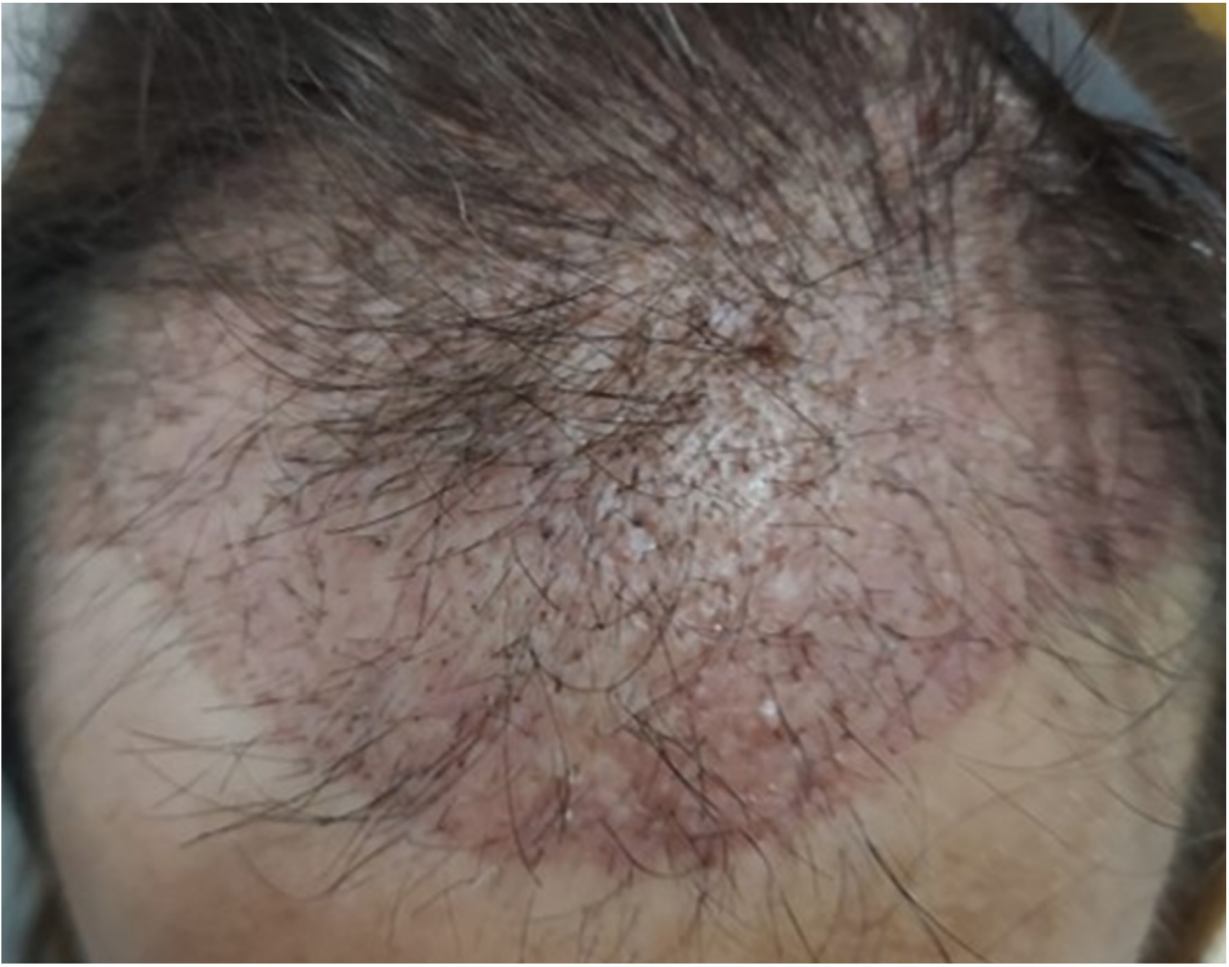
Recipient-site telogen effluvium.

Clinically, recipient-site effluvium manifests as diffuse or patchy shedding of pre-existing terminal hairs within or adjacent to the recipient area (Figure 9). Mir-Bonafé et al. (71) recently described an early-onset linear variant of recipient-site effluvium associated with dense packing. Although typically temporary, a permanent reduction in native hair density may occur, particularly in follicles nearing the end of their growth cycle or already miniaturized (12).

Management is primarily conservative, focusing on patient reassurance and expectation setting. Patients, particularly women, should be informed preoperatively about this possible transient shedding. Topical or oral minoxidil may help accelerate regrowth, while cosmetic camouflage (fiber sprays or powder concealers) can improve appearance during recovery (12). Preventive measures include avoiding excessive implantation density, limiting tumescence, minimizing local trauma and ischemia through gentle technique, and avoiding deep or wide incisions that may damage native follicular units.

#### 3.3.6 Unnatural results

**Figure 10:**
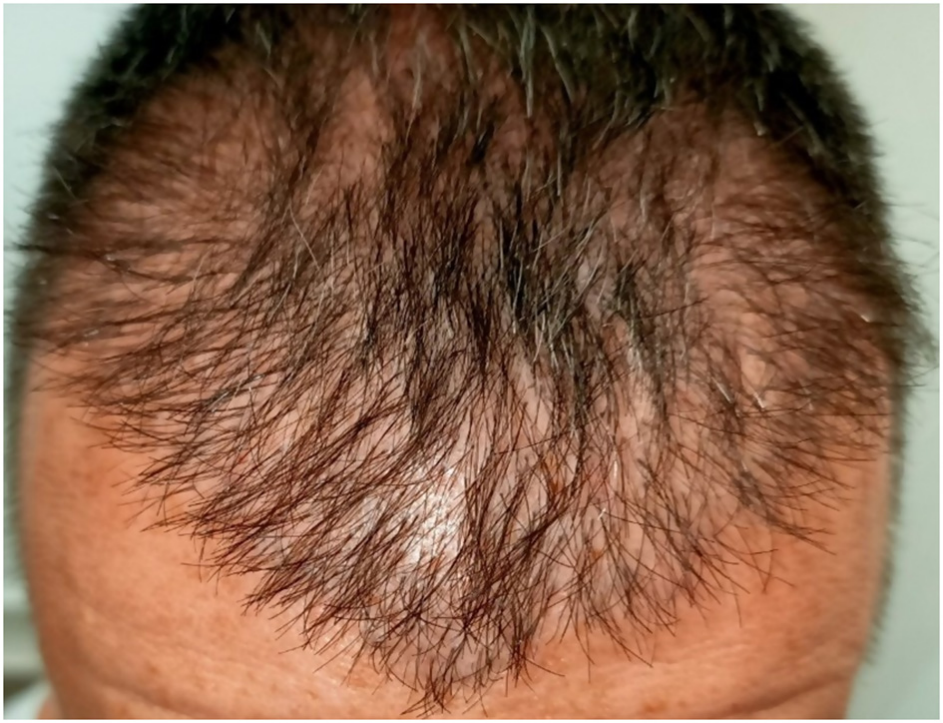
Unnatural appearance due to inaccurate graft angulation and insufficient density.

**Figure 11:**
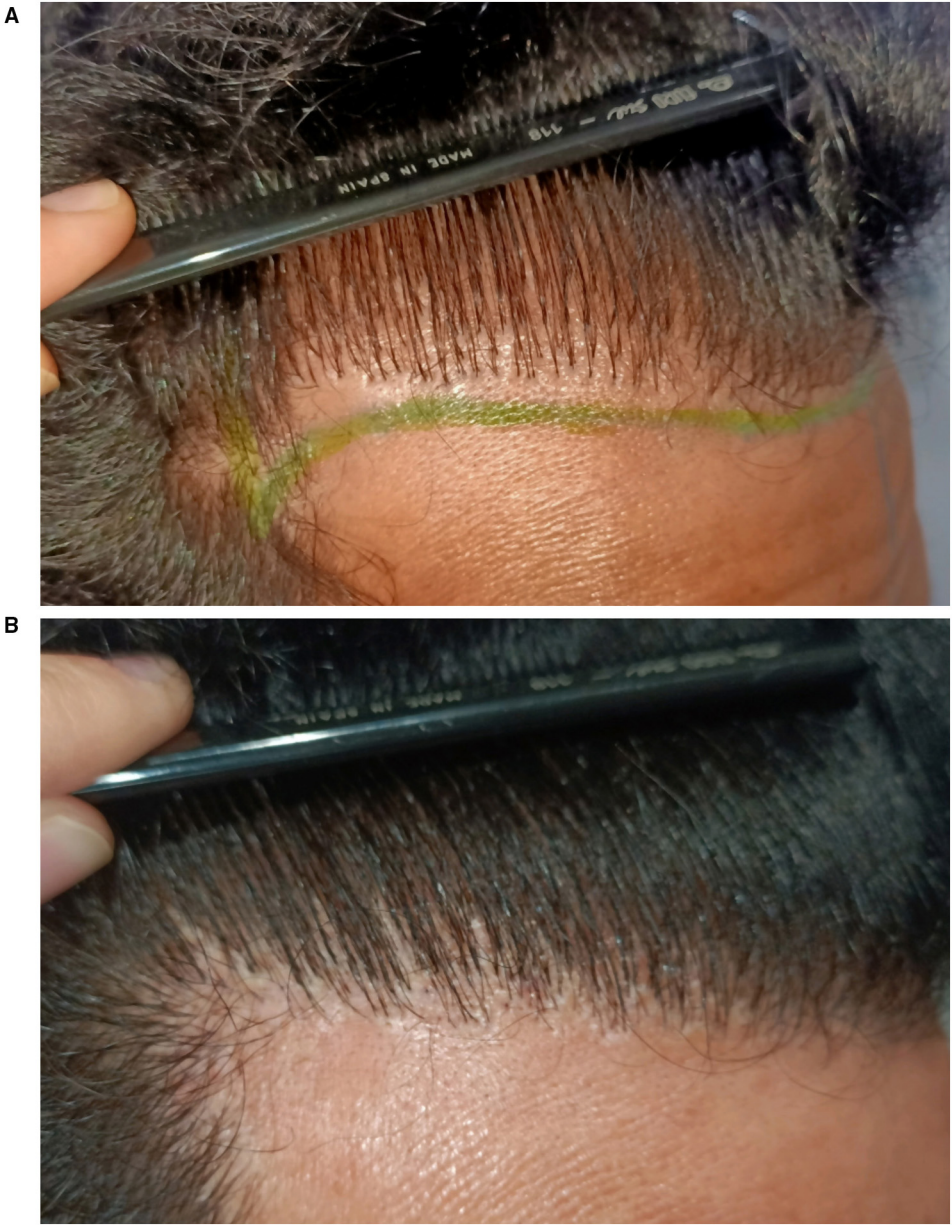
**(A)** Unnatural “pluggy” frontal hairline due to the use of multi-hair grafts in the first line **(B)** Correction of the frontal hairline by adding single-hair follicular units to create a softer, more natural transition.

Unnatural results represent one of the most frequent aesthetic complications after hair transplantation and are a major cause of patient dissatisfaction. The primary cause is iatrogenic, most often resulting from inadequate preoperative planning or lack of surgical experience. These outcomes may result from improper hairline design, inaccurate graft angulation, direction (Figure 10), or height, irregular density distribution, or the use of large multi-hair grafts in the frontal region, which can create an unnatural “pluggy” or tufted appearance (Figure 11) (1, 3, 9, 12). Low graft survival, poor donor-recipient matching, and visible scarring can further compromise the cosmetic outcome.

The naturalness of a transplant depends not only on graft growth but also on the ability to reproduce the patient's original follicular orientation, density gradients, and temporal symmetry. The frontal hairline is particularly critical: abrupt, sharply defined lines or symmetric patterns tend to appear artificial. Excessive density in the anterior transition zone or inconsistent spacing between grafts also disrupt the visual flow of the hair (1, 9). Poor outcomes may also result from inadequate anticipation of future alopecia progression or insufficient adherence to medical therapy, leading to disproportionate loss of native hair relative to transplanted grafts.

Prevention relies on thorough preoperative evaluation and patient counseling. The hair surgeon should clearly discuss the overall surgical goals, donor limitations, and the anticipated progression of androgenetic alopecia to establish realistic expectations (1). A gradual, irregular hairline design, placement of single-hair grafts in the frontal zone, and consistent control of graft angle and direction are essential to achieve natural results. Careful graft handling and depth control further optimize survival and minimize pitting or elevation.

Management of unnatural results focuses on aesthetic revision. Techniques include surgical excision or removal of misplaced grafts, redistribution, or camouflage by adding single-hair follicular units in the affected area. In selected cases, laser hair removal or electrolysis can be employed to soften excessively dense or misplaced grafts (1, 9).

#### 3.3.7 Poor growth

Poor graft growth is most often attributed to physical damage of the follicular units during harvesting, handling, or implantation (1, 6, 12). Follicular injury compromises the viability of the dermal papilla and bulge regions, both essential for regeneration and subsequent hair growth.

During extraction, various forms of trauma can occur. These include dermal sheath rupture (paring), follicular fracture, partial or complete transection, which should remain below 10%, and ideally between 1 and 3% in experienced hands (6). Other recognized extraction-related injuries are graft slippage, entrapment, tethering, capping (decapitation of the follicular tip), iatrogenic splaying (graft divergence), and denudation (de-epithelialization).

Additional manipulation injuries occur during graft handling and placement. Excessive pressure from forceps or implanters may result in crushing of the bulge or dermal papilla, while prolonged out-of-body time and inadequate hydration cause dehydration and desiccation, leading to irreversible cellular damage (6).

Patient-related conditions, such as smoking, diabetes, vascular disease, or preexisting scarring can also impair healing and may reduce graft survival (12). Suboptimal donor characteristics, including low follicular density or fine hair shafts, can limit perceived density even when graft uptake is adequate. Even when optimal technique is observed, a minority of patients (0.5%−1%) may exhibit poor growth due to an idiopathic mechanism known as factor X, likely related to interindividual variability in scalp vascularity or intrinsic follicular growth potential (37).

Prevention is primarily technical. Key measures include minimizing out-of-body time, maintaining constant graft hydration in chilled isotonic solutions, gentle graft handling, using appropriate instruments, employing a precise punch technique with correct angulation and depth, and avoiding excessive implantation density or deep slit creation (37). Consistent team training and surgical magnification further reduce transection rates and improve long-term graft survival.

#### 3.3.8 Inflammatory and autoimmune reactions

Autoimmune and autoinflammatory reactions are rare complications following hair transplantation but may have significant clinical implications due to their potential to cause irreversible graft loss. The most frequently reported autoimmune reaction is lichen planopilaris (LPP), although rare cases of erosive pustulosis of the scalp (EPS) have also been described.

LPP after transplantation appears to be uncommon (72–75), and several reported cases may represent previously unrecognized disease rather than true *de novo* onset triggered by surgery. Clinically, post-transplant LPP typically manifests months to years after transplantation with progressive perifollicular erythema, symptoms such as pruritus or burning, and gradual loss of follicular openings in the recipient area. The proposed mechanism involves trauma-induced immune activation through the Koebner phenomenon, leading to collapse of follicular immune privilege and a lichenoid lymphocytic attack on hair follicles (74, 75). Histopathology reveals a perifollicular lichenoid lymphocytic infiltrate with follicular interface damage and variable concentric perifollicular fibrosis, indistinguishable from idiopathic LPP.

Importantly, postoperative inflammatory changes must be carefully distinguished from true LPP. Alcântara et al. (76) demonstrated that perifollicular lymphocytic infiltrates and fibrosis can be observed in clinically normal scalp after uncomplicated hair transplantation, even in the absence of clinical signs of cicatricial alopecia, reflecting postoperative immunologic remodeling rather than active LPP. Likewise, trichoscopy of normally growing transplanted hairs may show perifollicular hyperkeratosis or erythema that can persist for several months after surgery; these findings are nonspecific and, in isolation, should not be interpreted as diagnostic of LPP.

For this reason, scalp biopsy should not be performed routinely in cases of poor growth or nonspecific postoperative inflammation. Histological evaluation should be reserved for situations with a high level of clinical suspicion, such as the appearance of new or progressive perifollicular erythema accompanied by symptoms (pruritus, pain or burning) and objective loss of follicular openings, particularly after an initial period of satisfactory graft growth.

EPS has also been described after transplantation, occurring in both younger and older patients (75, 77). It typically presents with pustules and crusted erosions in the recipient area that heal with scarring alopecia. Its pathogenesis is believed to represent a nonspecific inflammatory response to local trauma (75).

Although uncommon, these entities are clinically relevant, as they may result in permanent loss of transplanted hair. Careful preoperative clinical and trichoscopic evaluation is essential to exclude subtle or early forms of cicatricial alopecia before surgery, ensuring appropriate patient selection and reducing the risk of misdiagnosis (74, 75). When diagnosed, management should be based on the underlying condition and follow disease-specific therapeutic recommendations.

## 4 Discussion

Follicular unit excision (FUE) is widely regarded as a safe and effective surgical technique for hair restoration, although, like any surgical procedure, it is not entirely exempt from complications. The present review summarizes and discusses the current literature on adverse events associated with FUE, providing an updated overview of their frequency, underlying mechanisms, preventive strategies and management. From a clinical perspective, most complications are mild or transient, although certain events may compromise graft survival, aesthetic outcomes, or patient satisfaction if not properly recognized and managed. As the popularity of FUE continues to rise worldwide and procedures are increasingly performed in high volumes and by surgeons with variable training, the careful analysis of complications has become even more relevant.

Recent retrospective series and systematic reviews have confirmed the overall safety of modern hair transplantation, with most studies reporting low rates of adverse events. Liu et al. (4) estimated an overall complication rate of approximately 1%−5%, although these data were derived from historical series using mainly the FUT technique. More recent evidence focused on FUE confirms comparable safety outcomes. In a retrospective study of 1,030 FUE procedures, Aksoz et al. (8) reported notable complications in 9.9% of cases; however, nearly half of the patients experienced mild postoperative events, most commonly slight frontal edema and donor-site pruritus. In their systematic review, Khatib et al. noted that differences in surgical technique directly influence complication rates: although FUE may offer a slightly better safety profile than FUT, this has not been statistically confirmed. The authors emphasized that proper preoperative assessment, surgical experience, and meticulous technique remain essential to minimize complications (2).

Donor-site complications such as pain or sensory alterations are among the earliest and most frequent postoperative events. These typically result from transection of small cutaneous nerves during multiple punch excisions and are usually transient, resolving within weeks or months. In rare cases, neuropathic pain or dysesthesia can persist for longer periods. Other complications, such as bleeding, hematoma, or visible scarring, have become uncommon with the use of micro-punches and atraumatic techniques, although excessive harvesting can still lead to donor depletion, textural irregularities, or “pinpoint white dots” that may be visible in patients with short hair. In recent years, several authors have warned about an increasing incidence of iatrogenic injuries and poor outcomes related to inadequate training or the delegation of key surgical steps to unqualified personnel, highlighting the importance of maintaining strict professional and technical standards.

Recipient-site complications account for most clinically relevant postoperative problems. Folliculitis, perifollicular erythema, and persistent crusting are among the most frequent findings, generally reflecting localized inflammation or delayed healing. In addition, temporary effluvium of native hairs may develop as a multifactorial response to surgical trauma, inflammation, or transient vascular compromise. Although this shedding is self-limited, it can temporarily affect the cosmetic appearance and raise patient concern, particularly in women or in cases of dense packing. Awareness of this phenomenon and appropriate preoperative counseling are essential to prevent misinterpretation as graft failure.

Necrosis at the recipient site remains exceptional but clinically significant, typically associated with excessive density, deep incisions, high tumescence, and patient-related predisposing factors. The current trend toward higher implantation densities may predispose to smaller, multifocal, and more superficial ischemic areas, with a lower overall impact on graft survival compared with the larger single necrotic plaques described in earlier reports. Early identification of ischemic changes and conservative management are essential to prevent decreased graft survival and scarring.

Autoimmune reactions such as lichen planopilaris, although rare, may also emerge in the postoperative period and can be difficult to distinguish from normal postoperative inflammatory remodeling on trichoscopy and histopathology, even for experienced dermatopathologists. Careful preoperative clinical and trichoscopic evaluation is therefore essential to exclude preexisting disease. Postoperative assessment should rely on clinical evolution rather than isolated trichoscopic or histologic findings, and LPP should be considered only when symptoms or progressive loss of follicular openings develop.

In addition to these medical complications, suboptimal aesthetic outcomes such as poor growth, asymmetry, or unnatural hairline or direction may also occur, particularly in cases of inadequate surgical planning, inexperienced operators, or unbalanced density distribution between donor and recipient areas. Although not life-threatening, these outcomes have a major impact on patient satisfaction and the perceived success of surgery.

Based on current evidence, several preventive measures can help minimize these risks. Careful patient selection, smoking cessation, and optimization of comorbidities are essential preoperative measures, together with comprehensive counseling to ensure realistic expectations and adherence to postoperative care. During surgery, minimizing tumescence, using low concentrations of epinephrine, and avoiding excessively high implantation densities (< 45–50 FU/cm^2^) can reduce ischemic complications. Gentle graft handling, continuous hydration, and prevention of desiccation or prolonged ischemia are equally important to preserve follicular viability. Early postoperative washing, good hygiene, and prompt treatment of inflammation or infection help prevent secondary damage, while patient education about the transient nature of erythema, crusting, and shedding improves understanding of the recovery process and enhances overall satisfaction. Close postoperative follow-up further supports early detection of complications and optimizes outcomes.

In recent years, perioperative regenerative adjuncts have gained increasing attention as supportive strategies to optimize postoperative outcomes following FUE. Platelet-based therapies, including platelet-rich plasma (PRP) and platelet-rich fibrin (PRF), have been proposed to enhance wound healing and modulate postoperative inflammation, with potential benefits for graft survival and a reduction in postoperative shedding or recipient-site erythema (78–82). Systematic reviews and meta-analyses in androgenetic alopecia have demonstrated improvements in hair density and thickness with activated PRP, supporting its perioperative use in hair transplantation, although transplant-specific evidence remains limited (83). Low-level light therapy (LLLT) has also shown beneficial effects on follicular cycling, microcirculation, and inflammatory modulation, and is increasingly used as an adjunctive or maintenance therapy after hair transplantation (79, 84–86). Overall, while these approaches reflect current clinical trends, high-quality prospective studies are still required to define their true impact on postoperative complications and graft survival.

The current literature on FUE-related complications presents several methodological limitations. Most available studies are observational and predominantly retrospective and single-center, with heterogeneous definitions of complications, variable follow-up durations, and a lack of standardized reporting. In addition, many reports do not clearly differentiate between FUE and FUT or fail to provide detailed analysis of technique-specific parameters such as punch size, device type or implantation method. Underreporting represents a further limitation, particularly for mild or self-limited complications. Smaller series and case reports often describe a broad spectrum of adverse events, whereas larger cohorts tend to focus on severe or unusual complications, potentially underestimating the true frequency of common postoperative events. Relevant confounders, including patient demographics, comorbidities, perioperative medication use, anesthesia technique, and surgeon experience, are also inconsistently reported or insufficiently analyzed.

As a narrative review, the present work is inherently limited by the quality, heterogeneity, and reporting standards of the existing literature, which restricts the ability to draw firm conclusions regarding true incidence rates or causal relationships. The absence of standardized definitions and uniform reporting systems continues to hinder meaningful comparison across studies and the development of robust, evidence-based preventive strategies.

Given these limitations, future research should prioritize the development of standardized definitions and grading systems for hair transplantation-related complications, enabling consistent reporting and meaningful comparison across studies. Multicenter prospective studies and well-designed clinical trials are needed to better define the true incidence, risk factors, and clinical impact of FUE-related complications. In addition, collaborative registries and long-term follow-up data would help clarify the influence of patient-related, technical, and procedural variables on graft survival, postoperative safety, and aesthetic outcomes, ultimately strengthening the evidence base for prevention and management strategies.

Recent expert consensus statements have attempted to address some of these gaps by proposing structured recommendations for preoperative assessment, intraoperative technique, and postoperative care, with the aim of improving reproducibility and safety (80, 87). However, evidence directly linking specific intraoperative variables, such as ischemia time, graft hydration medium, or handling techniques, to clinical outcomes remains limited.

Emerging research directions are focusing on novel regenerative and technological strategies aimed at further optimizing graft survival and procedural safety in FUE. Exosome-based therapies are being explored for their potential to modulate inflammation, enhance angiogenesis, and support follicular regeneration; however, evidence supporting their role in hair transplantation remains preliminary and largely experimental (88–90). In parallel, technological innovation is advancing rapidly, with artificial intelligence-assisted systems emerging as valuable tools for surgical planning, intraoperative guidance, and postoperative evaluation, while robotic platforms being refined to enhance precision and reproducibility in FUE procedures (91–94). Together, these innovations hold promise for improving graft survival, surgical accuracy, and overall safety, potentially contributing to a reduction in postoperative complications.

## 5 Conclusion

Despite its minimally invasive nature, FUE hair transplantation is not devoid of complications. Although most adverse events are mild and self-limited, some may affect graft survival, aesthetic outcomes, or patient satisfaction if not properly anticipated and managed. Careful patient selection, individualized surgical planning, meticulous technique, and structured postoperative care remain essential to minimizing risks and achieving optimal results.

This review highlights the need for greater standardization in terminology, reporting, and perioperative protocols to enable more consistent assessment of complications and outcomes. Future multicenter studies, supported by consensus-based definitions, will be essential to establish evidence-based clinical guidelines. Together with the integration of emerging technological advances, these developments will be key to refining preventive strategies and further enhancing the safety of modern hair transplantation.
